# Disease control during livestock movement among smallholder farming actors in Kisumu West Sub-County, Kenya

**DOI:** 10.1016/j.vas.2026.100765

**Published:** 2026-07-10

**Authors:** Jackline Akoth Owili, Harro Maat, Barbara van Mierlo

**Affiliations:** Knowledge, Technology and Innovation (KTI) Chair Group, Department of Social Sciences, Wageningen University and Research, P. O. Box 8031, 6700 EW Wageningen, The Netherlands

**Keywords:** Livestock movement, Livestock health, Disease control, Social practice theory, Kisumu west sub-county, Smallholder farmers

## Abstract

Livestock movement remains a crucial socio-cultural practice for rural households in sub-Saharan Africa, yet disease-control practices during movement among sedentary smallholder farmers remain poorly understood. This study examined the relationship between livestock movement and disease-control strategies among smallholder farmers and other actors in Kisumu West Sub-County, western Kenya. Inspired by social practice theory and using a qualitative research design, data were collected through semi-structured interviews with 28 actors and two focus group discussions involving 16 participants, enabling data triangulation. The results reveal three distinct forms of livestock movement; intra-local, inter-local, and extra-local, each associated with specific disease-control challenges and responses. Farmers drew on locally available resources, community knowledge, and selective veterinary engagement to manage disease risks, often constrained by limited space, inadequate enforcement of health regulations, and economic pressure. Findings are context-specific and should be interpreted as qualitative insights from one sub-county rather than broadly generalisable evidence. The study offers implications for animal health governance in smallholder systems in developing countries like Kenya.

## Highlights

• Our study explored farmers' livestock movements in Kenya.
• Farmers understand their local disease ecology.
• Animal diseases are managed based on available local resources.
• Farmers prefer natural bull breeding to artificial insemination methods.
• Farmers face the high cost of animal health care and a delay in service response.

## 1 Introduction

In sub-Saharan Africa, the movement of livestock is deeply ingrained in rural life and farming practices as a prerequisite for feeding and trade in animals and animal products (Liao et al., 2020; Turner & Schlecht, 2019). However, livestock movement is not without risks. Various studies show that movement of livestock drives the spread and transmission of infectious diseases (Lelenguyah et al., 2024; Rivero et al., 2021). Whereas trade often implies movements over larger distances, farm composition and feeding practices create movements at a more local scale, for example, animals brought to harvested fields to eat crop residues or forage in communal spaces (Ekwem et al., 2023; Gizaw et al., 2021). This paper addresses the ways in which sedentary smallholder livestock farmers in Western Kenya balance feeding and other requirements of their animals, the conditions for trade in animals, and the disease risks these entail.

The role of livestock markets in disease spread, by bringing together animals from different locations, has been documented in various studies (Ekwem et al., 2023; Griffith et al., 2020; Lumborg et al., 2021; Mutua et al., 2019). In Eastern Africa, common preventable and curable infectious diseases including Foot-and-Mouth Disease, Rift Valley Fever, and Bovine Brucellosis reduce farmers' income and food self-sufficiency (Ekwem et al., 2021; Floyd et al., 2019a; Jahel et al., 2020). Literature points to two causes for the persistence of infectious diseases: a lack of access to animal healthcare services, often compounded by inability to pay and limited diagnostic capacity (Ogola et al., 2018; Tigoi et al., 2020); and adherence to cultural norms related to livestock, such as animals as gifts and shared use of grazing grounds and pens (Majiwa et al., 2022; Mutua et al., 2022; Omondi et al., 2021b).

Kisumu West Sub-County has favorable climatic conditions that support various farming practices because of its diverse rural farming communities (Ngigi et al., 2021). Livestock keeping in Kisumu West Sub-County is widespread across different socio-economic groups, including smallholder and female-headed households, where it acts as a primary income source and helps improve household resilience (Majiwa et al., 2022). In these livestock farming communities, mixed crop-livestock farming is common, with farmers usually keeping cattle, sheep, and goats, most of which are local and mixed breeds adapted to the local environmental conditions (Wahome et al., 2024). Despite the diverse nature of farming activities, livestock diseases are a persistent challenge to farmers in Kisumu. Studies show that diseases are a major constraint on productivity (Maree et al., 2025; Omondi et al., 2021b).In this region, herds commonly face Brucellosis, Lumpy Skin Disease, East Coast Fever, and Tick-borne diseases, which also pose significant challenges affecting most households in rural areas. At the same time, gaps in access to animal healthcare services exacerbate farmers' vulnerability to animal health threats (Mutua et al., 2019). In this paper, we analyse how smallholder farmers and livestock owners deal with such risks and how they manage diseases in the process of moving their animals.

Despite these documented risks, the disease-control practices of sedentary smallholder farmers during livestock movement remain underexplored, particularly in densely settled mixed-farming communities where land fragmentation has transformed traditional movement patterns. Kisumu West Sub-County provides an instructive case: land subdivision has replaced communal grazing with tethered, homestead-based management, yet animals still move for feeding, breeding, and trade. How farmers in this context manage disease risks across these different forms of movement is not well documented.

Our study focused exclusively on sedentary livestock farmers, those who manage animals from fixed homesteads, and the ways in which they manage animal health in relation to animal movements. We reference Kenya's pastoralist traditions only to contextualise the country's diverse livestock systems, not as the primary focus: animals from agropastoral, pastoral, and sedentary smallholder systems often converge in shared spaces such as markets and watering points, and these meeting points increase the potential for disease transmission. Climate change adds pressure by altering grazing availability and drinking water supply, with direct consequences for market movements in extreme cases (Maree et al., 2025; Ngigi et al., 2021).

To understand farmers' activities and decision-making regarding livestock movement, the study is inspired by social practice theory (SPT). This theory posits that human behaviour is best understood as contextual to the practice people operate in, consisting of three principal components: materials, competences, and meanings (Reckwitz, 2002; Shove et al., 2012). These components were used as analytical reminders rather than deductive categories: they helped us identify the resources farmers draw on (materials), the skills and knowledge they apply (competences), and the motivations and social norms that guide their decisions (meanings). This framing is particularly suited to the research questions because it directs attention to the situated, often informal logic behind farmers' movement and disease-control decisions, rather than treating these decisions as individual rational choices.

The objective of this paper is to examine livestock movements in relation to animal health as an inherent element of livestock management practices among smallholders in Western Kenya. We address two research questions: Why do farmers move their animals despite the disease-spread risks? And how do farmers deal with diseases before, during, and after animal movement? These questions are explored across three movement categories; intra-local, inter-local, and extra-local, which together capture the full spatial range of smallholder livestock movement in the study area. The subsequent sections describe the qualitative approach for data collection and analysis. The results illustrate how smallholder farmers' disease-control decisions are shaped by their needs, resource availability, and cultural obligations. The final sections discuss the findings and draw conclusions.

## 2 Materials and methods

This study was conducted in Kisumu West Sub-County, located in Kisumu County, Kenya, along the shores of Lake Victoria. This region was selected for its significance in mixed farming activities, including crop production, livestock farming, and cross-border animal trade. Kisumu West Sub-County mainly consists of smallholder mixed-crop farming, with most rural households divided into small units within and around their homesteads. These subdivisions are limited by small land sizes for household shelter and the integrated crop-livestock activities. The sub-county is positioned west of the Vihiga border, which serves as a regional livestock route for both Kisumu County and local markets such as Gambogi, Stendkisa, Kisian, and Mamboleo (Gerken et al., 2022). The study area included two locations: Nyahera and Sinyolo, both within Kisumu West Sub-County, each representing different sub-locations with multiple villages. In Nyahera, we conducted interviews in two villages: Karombo and Aluor. In Sinyolo, participants came from two villages: Kangasi and Buke. Overall, four village units were mapped for the study (Table 1). The study employed a qualitative study design to assess a case of how livestock farmers manage health risks associated with livestock movement practices.

**Table 1:** Categories of livestock and interview participants for each location.

Sub-Location	Village units	Livestock species	No. of animals owned per participant	No. of participants interviewed (*n* = 28)	
				participants without other income sources	participants with other income sources
Nyahera	Karombo	Cattle (cross)	3	4	0
		Goat (local)	5	2	0
		Sheep (local))	4	1	1 (Teacher)
	Aluor	Cattle (cross)	5	4	0
		Goat (local)	8	1	1 (Entrepreneur)
Sinyolo	Kangasi	Cattle (cross)	6	4	0
		Sheep (local)	4	3	0
	Buke	Cattle (cross)	9	1	1(Teacher)
		Goat (local)	3	2	1(Entrepreneur)
		Sheep (local)	3	2	0
			**50**	**24**	**4**
Total				**28**	

### 2.1 Participant selection and ethical clearance

We purposively selected participants with the selection criteria focused on farmers engaged in mixed farming, including their experience rearing local and crossbreed livestock such as cattle, goats, and sheep. These local breeds include animals raised over many generations to withstand harsh weather conditions; crossbreeds combine exotic and local breeds to improve survival and productivity. Participants also grew crops like maize, beans, and other legumes that serve as supplementary feed for animals. Their understanding of common livestock diseases, such as Rift Valley fever, anthrax, and brucellosis, along with their strategies for managing these diseases, was also considered (Campbell et al., 2020).

At the village level, initial contact was facilitated by village leaders and extension officers, who provided lists of eligible farming households. To minimise potential gatekeeper selection bias, the risk of directing researchers only toward more prominent or compliant farmers, we independently cross-checked these lists against informal preliminary visits to households in each village before finalising participants (Chenais & Fischer, 2018). This process helped ensure that participants included smallholders from varied socio-economic backgrounds and not only those with the highest visibility or social standing. Among the participants interviewed, a few were women working in different professions: two individuals are entrepreneurs and two are teachers, which provided an additional source of income alongside livestock farming. Interviews were conducted in various settings, including livestock markets, farms, and households.

The focus group discussion (FGD) participants were recruited separately from the 28 interview participants and there was no overlap between the two groups. The FGD participants were identified from individuals living in the study area who owned livestock, had experience managing livestock diseases on their farms, and had not already participated in the individual interviews.

Ethical approval for this study was obtained from the National Council for Science, Technology, and Innovation Committee (NACOSTI/P/23/25,811) and the Institutional Ethics and Review Board of International Livestock Research Institute (ILRI), Kenya (ILRI-IREC2023–52). We sought permission from the local village administration to conduct the study. Written consent was obtained from participants who could read and write; verbal consent was obtained from illiterate farmers, for both the semi-structured interviews and focus group discussions. Throughout the study, participants were allowed to withdraw at any time. The characteristics of participants are outlined in Table 1 for the interviews and Table 2 for the FGDs below.

**Table 2:** Characteristics of the focus group participants recruited for the study.

FGD group	Gender	Farmer category and livestock owned	Livestock diseases experienced	Meeting location	No. of participants
Group 1	3 Male 5 Female	Cattle, goats, and sheep	Anthrax, foot and mouth disease, brucellosis, tapeworms	Nyahera community center hall	8
Group 2	4 Female 4 Male	Cattle and goats	East coast fever, Riftvalley fever, lumpy skin disease	Sinyolo Chief’s camp social hall	8
				**Total**	**16**

**N/B:** The category of farmers in the table refers to livestock farmers rearing different animal breeds.

### 2.2 Data collection and analysis

Participants were invited for semi-structured interviews and focus group discussions (FGD). Interview guides featured open-ended questions that explored the reasons for livestock movement, health and biosecurity practices linked to movement, and changes in livestock movement trends. To understand the material aspects influencing farmers' decisions regarding livestock movement, for example animal feeding, treatments administered, and specific grazing locations, we probed the competences of smallholder farmers by asking how they monitor illness during and after moving livestock and where they seek information about livestock movement. We also asked about the reasons and motivations (meanings) guiding their decisions to move animals to particular locations. Participant observation involved taking field notes on farmers' daily feeding routines. Semi-structured interviews were used to gain insights into farmers' criteria for selecting specific livestock routes for grazing or going to livestock markets.

All semi-structured interviews were conducted in Dholuo (the local language) or in English, depending on the participant's preference. Transcription from Dholuo to English was conducted verbatim by the first author and two trained bilingual research assistants. To verify translation accuracy, a sample of transcripts was back-translated into Dholuo by a research assistant who had not participated in the original transcription, and discrepancies were resolved through discussion among the research team.

Two focus group discussions (FGDs) with sixteen selected livestock farmers and owners were held (Table 2). The FGD participants were different from the 28 interview participants, with no overlap between the two groups. Both groups included male and female farmers who raised at least one of the three livestock species; cattle, sheep, or goats, and lived within the study area. Participants were chosen because they owned livestock, had knowledge of common livestock diseases affecting their herds, and were engaged in daily animal husbandry activities. The FGDs were conducted in Dholuo and were led by the first author with support from two trained research assistants. The two data collection methods, together with participant observation field notes, provided data triangulation, enabling cross-checking of findings across sources (Kumar Ranjit, 2023).

Interviews lasted 30 to 40 min, while the focus group discussions lasted about one hour. Both were audio-recorded. All transcripts in Dholuo were transcribed verbatim into English by the first author and research assistants, as described above; English-language interviews were transcribed directly. The analysis began with the first author familiarising herself with the translated interview transcripts and field notes, which were organised using ATLAS.ti (version 24) software. A thematic analysis approach was used to identify key themes from participants' conversations. First, the transcripts were read to identify key themes inductively, meaning themes were derived from the data rather than from predetermined ideas (Nowell et al., 2017). This aimed at identifying recurrent descriptions of livestock movement and disease-control activities, as well as daily herd management routines.

Initial coding captured participants' statements regarding veterinary support on farms, movement decisions, health permit regulations, feeding patterns, and treatment withdrawals (Naeem et al., 2023). In the second phase of coding, patterns emerged that expanded how participants differentiated their animal movement practices within homesteads (intra-local), between neighbouring villages (inter-local), and across regional boundaries or markets (extra-local). Themes were developed iteratively in the sense that after initial coding by the first author, the emerging themes were reviewed and discussed with the co-authors, and any disagreements about the categorisation of codes were resolved through consensus. Throughout coding, the research team remained attentive to the possibility of social desirability bias, which is the tendency for participants to report what they believed was the expected or correct practice rather than what they actually do, and interpreted participant statements accordingly, particularly where self-reported practices could not be independently verified. Data triangulation across interviews, FGDs, and field observation notes was used to assess consistency of key findings and to identify where self-reported accounts diverged from observed behaviour. These thematic codes are used to present the main findings in the next section.

## 3 Results

The results are structured into three distinct forms of animal movement: (3.1) intra-local, (3.2) inter-local, and (3.3) extra-local movement practices. In the following sections, we present these three types of animal movement associated with farmers' disease-control practices. Animals and livestock are used interchangeably, but they represent cattle, goats, and sheep.

### 3.1 Intra-local movements

**Fig. 1:**
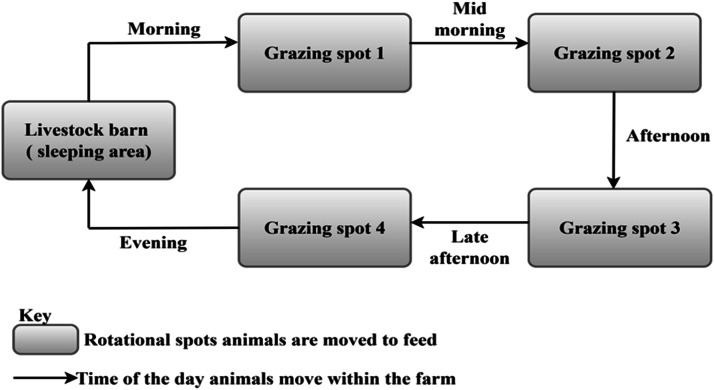
A flow chart of intra-local movement of animals. Some farmers use tethering method, some use paddocks, and others employ a herdsman to watch over animals as they graze to control movements.

Intra-local movement refers to animal movements limited within the homestead and adjacent plots, where animals from the same household interact. This is influenced by small land sizes and household labor. This form of movement can be broken down into three sub-forms that require different combinations of resources such as tethering spots, animal feeds (grass or crop residue), water, and animals. First, animals are moved from the barn early in the morning. Next, they are tied to a different spot mid-morning, where they begin feeding on crop residue or cut grass from local farms. After that, they are taken to a third spot in the afternoon and given fresh feed and water. Finally, they are tied to rest before being returned to the house in the evening, after sunset (Fig. 1). Cattle, goats, and sheep are kept within the homesteads without having to move outside the farmers’ compound. This form of movement is typical in sedentary livestock-rearing communities. A prominent change farmers experienced in the intra-local movement of livestock that emerged from the data is the near elimination of the traditional community practice of moving livestock through communal areas in search of pasture and water. Although daily movement is a core part of animal husbandry, most smallholder farmers now keep their animals within small distances from or on their homesteads. Managing this form of intra-local movement would involve farmers associating disease control with finding ways to keep animals fed and limit starvation. In other words, moving animals to graze has increasingly been replaced by transporting grass and other feed to where the animals are. However, various conditions still require animal movements, as explained in the next subsection.

The rise in livestock diseases, such as East Coast fever, and evolving land-use resettlement patterns is driven by population growth and land fragmentation. Typically, land is subdivided among family members, resulting in smaller plots and more scattered farms. This trend increases pressure, reducing available space for isolating sick animals and hindering rotational grazing, which previously helped break the cycle of diseases such as tick-borne and internal parasitic infections. These mostly affected cattle and sheep. Land fragmentation pressures have also led to increased interactions between animals on farms, creating opportunities for diseases to spread quickly. Although land reduction led to the spread of disease among animals and forced farmers to live in proximity, some farmers acknowledged that they kept each other informed whenever their animals fell ill. This way, they could limit the spread of diseases by sharing information about biosecurity measures, such as routine spraying of animals on their farms to prevent tick-borne diseases. The reduction of land has enabled farmers to observe one another’s ways of managing sick animals during disease breaks on their farms. Such experiences and advice also spread quickly among farmers in close proximity, thereby enhancing disease-control efforts. In an interview with a dairy farmer managing a mixed herd of cattle, goats, and sheep on a quarter acre of land, he reflected on the differences between his current way of keeping livestock and how things were handled when he was young, and the related health risks. *“In the past, we moved our cows to different spots on the farm to rest after grazing, allowing the grass to regrow before returning the animals to eat the fresh grass. This method helped control internal parasites like worms, ticks and other ground infections. However, with smaller plots of land and increasing neighborhood resettlements, the animals are now tethered in the same spots year-round, and such diseases persist. The good thing is that, as neighbors, we notify each other on what to do when our animals are sick so that people can take extra disease control measures*.”

Furthermore, land fragmentation is not just about the physical spaces available for managing animals, but it also creates an environment where disease control becomes a socially negotiated practice. Instead of relying solely on formal veterinary guidance, farmers draw on their situated knowledge and practical experience to manage the small, fragmented plots and dispersed areas. In these spaces, farmers regularly demonstrate their skills through spontaneous and ongoing decisions, engaging in collective negotiations with neighbors about when to implement disease control measures.

Another farmer pointed out: *“Back in the days, the cows and goats would go far to graze, even in the forest. If either a cow or a goat falls sick, you could tie it to a tree and leave it behind to avoid it mixing with the healthy goats as you move on, then come back later to treat it before taking it back home. Nowadays, they feed from home, and if one gets sick, I can quickly isolate it; otherwise, the rest of the flock will follow one after the other”.* This farmer’s experience points to a key challenge of direct animal-to-animal contact, where animals are tied together to graze around the compound, making them vulnerable to health complications in the event of an outbreak. Farmers historically used simple methods like isolating sick animals in the forest to prevent disease spread, later returning to treat and reintegrate them into the herd. Today, animals are immediately separated from healthy herds once a disease is detected to monitor their health. This shows a shift away from a more communal solution (the forests) to individual control of disease spread within their farms.

This narrative was frequently shared among other households, especially by young farmers trying to manage good animal husbandry in small spaces. A young farmer, who had recently purchased improved dairy cattle breeds to test on his parents' land, faced a mix of opportunities and challenges. Standing by the entrance of the cattle shade, he said, *“ The vet tells us to keep the animal shade clean, spray animals for any ticks and administer vaccines, but the space is too small I tie the cows the entire day, they have wounds around their necks where the rope touches the skin, they do not feed well and sometimes these animals end up fighting and injuring themselves. If one has East Coast Fever, I can easily lock it in a small shade to prevent the whole lot of animals from being affected before you know it.”* These sentiments build on the idea of the old farmer above, where animal enclosures are now physically smaller but still allow for consistent observation of changes in animal behavior that may indicate illness. The reduced spaces play a crucial role in disease detection and intervention. In the effort for the young farmer to adopt improved animal husbandry practices such as semi-zero grazing and seeking veterinary support, the farmer is able to navigate the same system (zero-grazing) to minimise cross -transmission of diseases such as East Coast fever in cattle and sheep. Here, the animals’ health is constantly monitored, and they are confined to their shelter, which limits their movement. For the young farmer, this would imply turning the animal shelter into a space for treatment and quarantine, reducing the risk of disease spread.

The discussions with farmers indicate that intra-local movement of animals generally remains a confined activity within their immediate homesteads. This on-farm movement suggests that disease management is largely limited to the compound itself. The small, limited movement functions as a natural barrier, reducing the risk of transmission from neighbouring farms. Farmers reported that confinement makes disease detection and isolation simpler, as animals can be observed daily in a controlled space. However, zero-grazing and tethering also introduce distinct internal risks. Crowded enclosures, insufficient space for isolation, rope wounds that serve as entry points for infection, and the inability to rotate grazing areas all increase the risk of animal-to-animal contact and the persistence of parasitic and bacterial infections within the homestead. Disease dynamics in intra-local movement are therefore not simply self-contained in a protective sense, because confinement reduces some external transmission pathways while potentially intensifying others. Farmers' practices for isolating sick animals and maintaining clean enclosures, while reported as routine, were primarily self-reported and could not always be independently verified during field visits.

### 3.2 Inter-local movements

**Fig. 2:**
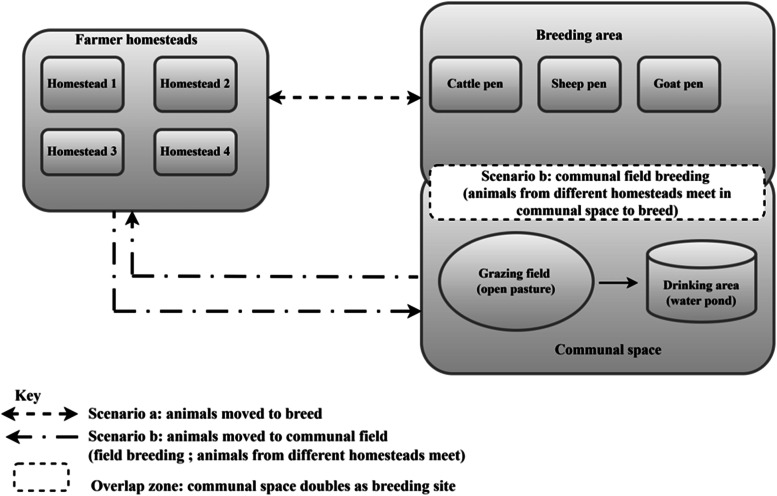
A flow chart of inter-local movement of animals.

While resource constraints and land fragmentation keep many animals tethered within the homestead for basic feeding (intra-local), economic and reproductive necessities regularly push other livestock into daily external movement environments (inter-local). Inter-local movement is a form of animal movement that occurs within and between fields and homesteads, where cattle, goats, and sheep spend much of their time outside the immediate compound. Usually, livestock farmers and herders move animals outside the homestead daily to graze on shared pastures, drink from communal water sources, forage along roadside fields, and return home in the evening. They repeat this cycle, which increases contact across these areas. The data revealed two main drivers in the animals' daily movements in this theme: searching for food and water for drinking, as well as sexual reproduction/breeding (See Fig. 2).

A first reason for interlocal movement of livestock is searching for feed. This practice is complicated by unpredictable seasonal changes, which are a key resource factor in determining the availability of animal feeds. Farmers frequently raised concerns that they have no alternative feeding options and thus rely heavily on moving their animals to graze in open fields. Several farmers lamented that pasture and water were essential to ensure the animals were well-fed during the dry periods, especially when feeds around the homestead were depleted. A farmer with indigenous cattle said: “*There is no grass or water here to feed my cows now because of the dry conditions. I have to take them to the riverbank so they can eat and drink to avoid starving.”* This statement was widely echoed and reflects a sense of responsible animal husbandry, prompting farmers to increasingly depend on alternative pastures to ensure their animals survive irregular climatic conditions. Thus, environmental conditions and alternative feeding spaces significantly influence how farmers can move their animals to access food and avoid starvation. Farmers' reliance on their understanding of the local ecology and climate informed their decisions regarding livestock inter-local movements.

A second reason for interlocal livestock movement is in relation to sexual reproduction. Farmers mentioned their reliance on the bull as a livestock species to serve multiple cows within their homesteads. In peak mating seasons, farmers frequently monitor cows by listening for their continuous “mooing sounds,” which signal the need to take the animal to nearby farms for mating. Farmers also mentioned that it is often difficult to find a healthy, mature bull. Farmers’ explanations about breeding show their competence in ‘listening’ to the cow’s conditions and deciding when and where to go for mating. Despite the health risks associated with shared bull use, it helps reduce breeding costs. A farmer with dairy cows stated: *“ I understand that bulls can transmit diseases sexually, such as vibriosis, but what options do I have? I can't afford artificial insemination, and most people don’t own bulls. I monitor the behavior of cows, like when they make funny ‘mooing noise’ during certain times and look for a mature bull, which I might find in the neighboring village in cases of injuries.”*

In relation to moving animals for breeding, farmers handled the use of bulls with caution to limit the risk of spreading disease. An important aspect that emerged in the group interviews was a concern about controlling disease transmission between animals and maintaining herd health. In the group discussions, many farmers expressed their preference for controlled bull mating practices, such as using a known bull within the community, and consulting a health service provider (veterinary officer) to cross-check if both the bull and the cow are clinically healthy for mating. An example quote from a group discussion indicated: *“The veterinary officer told us that using bulls to serve animals without knowing where they came from is not good because they can carry diseases, so we call the vet to come and inspect the animals first.”* The availability of healthy bulls and the presence of a veterinary officer reflect on farmers’ reliance on locally available resources at their disposal to perform safe reproduction practices to avoid the potential spread of diseases within their farms. The practice also shows that farmers are aware of direct animal-to-animal contact through breeding as a route of disease transmission. However, due to limited availability of bulls and associated costs, farmers continue to rely on bull serving that is locally owned within nearby farms, and they recognize the potential health risk, yet the cost alternative remains a constraint to them, hence using the help of a veterinary officer to assess the bull's overall health before mating. It should be noted that farmers' accounts of consulting veterinary officers before each mating event reflect a reported norm rather than a uniformly verified practice. Given the acknowledged limited availability of veterinary services in the area, such consultations may not occur as consistently as described. Controlled breeding with bulls holds social importance for farmers, as they select bulls from reputable owners. As such, this practice enables farmers to trace the origin of suspected disease for contamination measures. This sentiment about relying on bulls to serve the entire community highlights a longstanding tradition of maintaining animal lineages, despite the health risks of direct contact with animals.

### 3.3 Extra-local movements

**Fig. 3:**
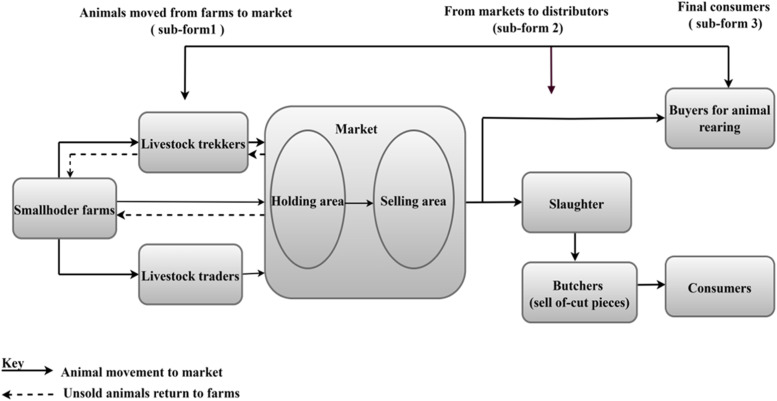
A flow chart of three sub-forms of extra-local movement of animals.

Extra-local movements of animals and animal products for other than reproductive purposes is mostly regulated by markets. These movements generally involve animals switching between owners. This can be either for continuing to live with a different owner or being butchered and ending up as meat sold in butcheries. Also, dairy and hides are part of these transfers. Not all animals that are moved for sale get new owners. When no deal is made, cattle, goats and sheep return to their original location (Fig. 3). Moreover, farmers often lend their animals to friends to rear them on their behalf. Lending animals to friends to rear is viewed as a cultural obligation to preserve longstanding kinship ties and reflects how social capital is a valuable asset for pooling risks in the event of a disease outbreak.

Moving cattle, goats, and sheep from one farm to another involves various additional actors, most prominently trekkers, traders and vets. Livestock trekkers – individuals who walk and control the animals as they move - typically consult with farmers about the health conditions of animals that are from different local areas. These trekkers are normally not the owners of livestock (cattle, goats, sheep, etc.). These concerns focus on selecting particular breeds or individual animals that can withstand weather changes and the associated health risks that arise when they are transferred to new owners. This is explained from a focus group discussion by a hybrid goat farmer in the following quote: *“I have a network of livestock trekkers that I usually contact when my goats give birth to many kids that I cannot keep in my small space. I can then send the trekker to walk the young goats to my friend's place near the Gambogi area, who checks for any health issues by calling me via phone when the goats are delivered to her. I know that the area has good weather for the goats to survive, in case of any outbreak. This provides me with a backup option for safeguarding my livelihood. About three years ago, all my goats died due to an outbreak. Nowadays, I do not take chances”.*

In some cases, implementing biosecurity measures appeared impractical, especially when animals or animal-sourced products are sold from farms to markets. Farmers faced difficult decisions of either selling and risking losing income by not adhering to the health permit requirements or forgoing sales to ensure compliance. While discussing the desire to move animals from farms to markets, farmers stressed that health clearance processes are crucial for verifying the health condition and ownership of their animals in long-distance movement. Veterinary support and health permit clearance are essential for regulating market access for animals and the sale of animal-sourced products, as well as for mitigating health risks in shared environments. Even though health permits are not strictly enforced, farmers find it simpler to move their animals freely for health verification. Nonetheless, they still need to report specific symptoms, including visible injuries and flu-like signs, to health service providers. However, the farmers interviewed noted that they frequently disregard health service providers' recommendations about reporting health issues in their animals, occasionally even neglecting advice against selling sick animals at the market. A dairy farmer reported, *“I once treated my cows for mastitis and was uncertain about the milking withdrawal period. I contacted the vet, who advised me to wait five days before restarting milking and to discard any milk produced during that time to prevent selling contaminated milk. I did not follow the vet’s advice because I needed money to buy stationery for my child’s school supplies, so I milked the cow and sold it to others.”*

Another farmer reinforced the sentiments: *“…. at one point, I sold my three cows at a local market without providing any health documentation to indicate whether they were unwell. However, one of the cows had received various medications yet showed no improvement. Although I am obligated to notify the vet about any health issues so that I can be issued a health clearance form, I failed to do so. Wanting to get rid of them and not wanting to return them home, I assured the buyer that all the animals were healthy.”* These accounts illustrate that selling sick animals is not a reflection of farmers' expertise in circumventing regulations, but rather a consequence of economic pressure, weak enforcement of health regulations, and limited access to affordable veterinary care. When the cost of treatment is uncertain and the prospect of returning unsold animals home represents a financial loss, bypassing health documentation becomes a rational response to structural constraints rather than a deliberate competence. Consequently, they conceal crucial information from potential buyers, because of the additional costs of treating sick livestock if it is detected to be sick in the market, potentially resulting in returning the animal home without making any sales from the market.

Deworming is another biosecurity measure that farmers use when planning to move animals to seek markets. They mentioned that when an animal, like a cow or sheep lacks appetite, they typically buy deworming powder such as albendazole from agrovet stores and mix it with water for the animals to drink. Besides obtaining medication from agrovets, farmers shared using herbal medication as a typical practice in treating ailments such as foot rot on cattle or goat hooves. Other farmers reported using warm wood ash on cow hooves as a disinfectant for cleaning and healing wounds. In one of the groups, farmers remarked, *“….when cows stop eating, we purchase albendazole deworming powder from the agrovet store. After mixing it into their drinking water, they typically return to their normal eating habits within a day. Their skin also appears healthier, which matters greatly, particularly when we intend to sell them. Sometimes, we use herbal remedies like root tubers and cactus leaves mixed in drinking water to deworm the animals and prevent roundworms. We also use warm burnt wood ash to disinfect wounds on their hooves. Our forefathers utilized this method, and we continue to use it today.”*

Farmers mentioned that obtaining wood ashes as a herbal remedy and agrovet medications are important for treating and preventing illness in their animals. More than direct cures for diseases, these products enhance the condition of the animals, for example, the animal’s appetite and skin texture. The use of herbal and agrovet medical products embodies farmers’ application of their skills to use both traditional and biomedical products as a way to improve their animal health conditions. It also indicates farmers' interest in ensuring their animals are in good health, thereby generating more income when sold at the market.

After moving animals, especially new animals or those returning from the market, farmers reported implementing health measures such as isolation, vaccination, and record-keeping for diseases and treatments. Using available resources such as separate pens, they described closely monitoring animals' health and behaviour before reintegrating them into the main herd, and administering vaccinations against diseases like anthrax, foot-and-mouth disease, and lumpy skin disease. Farmers also stressed the importance of maintaining vaccination records to track diseases affecting their herds and to identify potential outbreak periods. A cattle farmer in a focus group discussion described these practices: "*…isolating new animals in separate housing allows for close monitoring of their health and behaviour for about ten days. This minimises the risk of spreading contagious diseases like foot and mouth disease or lumpy skin disease to existing herds, as new goats or cattle may not show illness signs immediately, despite seller claims of treatment. I keep detailed records and vaccinate new arrivals.*"

These post-movement accounts indicate that farmers are aware of formal animal health standards for preventing disease transmission. However, these practices, record-keeping in particular, were self-reported and may be subject to social desirability bias; it is possible that actual compliance is less consistent than reported. Nonetheless, farmers' familiarity with isolation protocols and incubation periods reflects a meaningful level of disease awareness within this community (Table 3).

**Table 3:** Summary of intra-, inter-, and extra-local movements, their drivers, disease risks, and control practices.

Category	Spatial scope	Main drivers	Disease-control practices	Key risks and significance
**Intra-local**	Within the homestead and adjacent plots; animals do not leave the compound	Land fragmentation; population pressure; zero-grazing adoption; daily feeding and watering routines	Tethering rotation across four daily spots; immediate isolation of sick animals; neighbour-to-neighbour disease alerts; routine acaricide spraying	Reduces external exposure but increases internal risks: crowding, tethering wounds, inability to rotate grazing. Disease dynamics are largely self-contained, making monitoring feasible but isolation space scarce
**Inter-local**	Between homesteads, communal pastures, roadsides, water points, and neighbouring farms	Seasonal feed and water scarcity; sexual reproduction via shared bull; cost of artificial insemination	Farmer "listening" for cow oestrus signals; selective use of known, reputable bulls; reported pre-mating veterinary checks; reliance on locally owned bulls to trace disease origin	Daily communal grazing and shared water sources create repeated animal-to-animal contact. Bull sharing is the main disease transmission route at this scale; vet consultations are reported but not consistently verified
**Extra-local**	Across sub-county or regional boundaries; livestock markets, slaughterhouses, and friends' farms	Income generation; trade; cultural obligation (lending animals to kin); market demand for meat, dairy, and hides	Use of livestock trekkers with health checks; deworming and herbal treatments before market; post-arrival isolation of new animals (∼10 days); vaccination records; reported health-permit compliance	Highest disease-transmission risk: animals mix across origins, health documentation is weakly enforced, and economic pressure leads some farmers to sell sick animals. Trekkers and traders can bypass official health checks, compounding spread risk

## 4 Discussion

It is essential to understand how livestock farmers manage their animals during intra, inter, and extra-local movements, and how these activities influence disease control practices on their farms. Our study shows that livestock farmers are aware of the health risks associated with moving their animals and the appropriate control measures within their context. As also confirmed by Lane et al. (2025), these factors should be considered in disease control strategies during animal movement. Livestock farmers are aware of the health risks associated with moving their animals and the appropriate control measures within their context and these factors should be considered in disease control strategies during animal movement (Lane et al., 2025). Our results highlight different types of movement of livestock and how this is embedded in practices that combine traditional practices of animal movement across grazing zones with zero-grazing systems in which animals are penned to a single spot. This transformation was due to land fragmentation and rising population pressures, which led to encroachment into communal-owned grazing areas and their conversion into areas for human resettlement. The encroachment of communal grazing areas increased contact between animals and humans, as animals are kept close together and fed within the homestead. The gradual shift toward a zero-grazing system minimised animals’ exposure to disease risks within confined spaces, such as East Coast Fever and foot-and-mouth disease. Likewise, studies across Africa have shown that the rise in human-animal interactions within shared spaces is primarily linked to spillover events of zoonotic diseases (Kamau et al., 2021; Ngigi et al., 2021). Despite the risk of disease spread, the social reordering of human resettlement led to increased disease control measures among livestock keepers living close to one another. Disease outbreaks were reported in real time whenever a case occurred on a particular farm, enabling timely intervention measures, such as the use of dewormers and vaccinations against parasitic infestation. In many livestock-keeping communities in Kenya, community leaders shared information about a suspected animal disease, thereby enhancing the message's credibility. Farmers trusted these leaders as information sources and were more likely to follow recommended disease control measures and to mitigate potential spread within the community (Kang’ethe et al., 2012). Other studies have observed that livestock farmers' disease control practices are primarily based on locally available resources, such as outsourcing pasture and providing watering for animals, to avoid direct contact with other animals in the community. Therefore, confining animals to zero-grazing systems helps control disease within the homestead, making contact tracing easier in the event of an outbreak (Novick et al., 2025; Omollo et al., 2023). It is important to note, however, that the shift toward zero-grazing does not straightforwardly reduce disease risk. While confinement reduces exposure to external disease sources and facilitates individual monitoring, it simultaneously increases risks linked to crowding, limited space for isolation, poor ventilation, wound formation from tethering, and the inability to rotate grazing areas that previously interrupted parasite cycles. The net effect on disease burden depends on husbandry quality within the enclosed space, which varied considerably across farms in this study.

In this study, livestock farmers consistently emphasized the importance of preserving genetic continuity when describing the practice of using bulls for natural breeding. The use of bulls for natural breeding was meant to ensure safe reproduction according to trusted community sources (Pauley, 2024). In Tanzania, similar to findings of this study, livestock farmers depended on natural breeding choices and considered the method a local and socially accepted practice, even though animals were exposed to sexually transmitted diseases (Kitole & Sesabo, 2022). Livestock farmers struggle to adopt artificial insemination as an alternative breeding method due to the associated costs or breeding and the risk of producing animals with inferior genetic traits. This further aligns with Ssekibaala’s et al. (2024) study in Uganda, which highlights the high costs of feeds and animal health care services that burden farmers’ bull breeding and health management challenges. Others, though, have said that caution ought to be exercised when using both natural and artificial breeding methods, and that animal health care support from verified veterinary officers should be sought to ascertain the associated health risks before breeding (Mekasha et al., 2024; Ssekibaala et al., 2024). Studies have demonstrated that natural breeding methods are still used, especially in indigenous animal breeds in Africa, and that there is a low uptake of artificial insemination among smallholder farmers (Washaya et al., 2019). It should be emphasised that farmers' reported practices of consulting veterinary officers before bull mating, alerting neighbours to disease, and maintaining vaccination records reflect normative accounts that may be influenced by social desirability bias, the tendency to report expected or approved behaviour rather than actual practice (Schatzki, 2019). The study did not have the means to independently verify these practices, and the absence of direct observation of many disease-control activities is a limitation to bear in mind when interpreting the findings.

The livestock farmers were well aware that moving animals into communal shared spaces contributes to the spread of diseases. However, due to the scarcity of feeding resources, they continue to engage in these animal movement activities to earn income and provide for their household needs. Implementing disease control measures in livestock-keeping communities can be complicated due to laxity in the enforcement of health regulations during animal movement (Omondi et al., 2021b). In another study conducted in Western Kenya, livestock are often transported without proper health documentation, quarantine, or vaccination, raising the risk of disease spread across borders (Tigoi et al., 2020). This negligence can cause animal disease outbreaks, which also threaten human health through the consumption of animal products. Additionally, poor enforcement of animal movement laws can undermine the credibility of health services and result in substantial financial losses from disease control costs. In line with previous studies, it was found that farmers face challenges notifying their health service providers (veterinary officers) of a suspected animal illness due to poor relationships with veterinary officers or no-shows who do not provide the required animal health services (Davis et al., 2021). Thus, implementing and strictly monitoring animal health regulations for cross-border and regional movements is essential to protect public health (Gerken et al., 2022; Omondi et al., 2021).

Several limitations should be acknowledged. First, the study is geographically limited to two sub-locations within Kisumu West Sub-County; findings should not be generalised to other regions of Kenya or East Africa with different ecological or socio-economic conditions. Second, the sample is qualitative and non-random (*n* = 28 interviews, two FGDs), which limits statistical representativeness. Third, participant recruitment was facilitated by village leaders and extension officers, introducing a risk of gatekeeper selection bias toward more prominent or compliant farmers; steps were taken to minimise this (see Section 2.1), but residual bias cannot be ruled out. Fourth, most findings rest on self-reported practices, which are susceptible to social desirability bias; farmers may have overstated compliance with biosecurity norms. Fifth, direct observation of disease-control practices was limited to participant observation of daily feeding routines meaning that the majority of practices described including vaccination records, veterinary consultations, neighbour notifications, were not independently observed. Sixth, reported diseases were not confirmed by veterinary or epidemiological diagnostics. These limitations mean the findings should be read as context-specific qualitative insights into farmer decision-making rather than as broadly generalisable evidence of disease-control effectiveness.

This study focuses on sedentary smallholder livestock systems, not on zoonotic diseases or pastoralist production as ends in themselves. References to diseases like foot-and-mouth disease, brucellosis, Rift Valley fever, and anthrax serve to illustrate that poor disease management at the farm level carries consequences beyond livestock productivity, including risks to human health. Similarly, Kenya's pastoralist traditions are referenced only to situate sedentary smallholder systems within a broader livestock landscape given that animals from agropastoral, pastoral, and sedentary systems regularly converge at markets, watering points, and shared grazing areas, creating transmission pathways that cross system boundaries. Effective disease management among sedentary smallholders therefore matters not only within the homestead but within this wider livestock ecosystem, where the veterinary officer remains a critical but currently underutilised link.

## 5 Conclusion

This study contributes to the literature on livestock movement and disease management in smallholder systems by documenting the situated practices through which farmers in Kisumu West Sub-County navigate the competing demands of feeding, breeding, trade, and disease control. The findings are context-specific qualitative insights and should not be read as broadly generalisable evidence of disease-control effectiveness. These approaches can help inform interventions that benefit livestock-keeping communities and raise awareness among farmers about using artificial insemination as an alternative breeding method, especially since natural breeding is not sustainable due to the limited number of animals a bull can serve and the risk of spreading animal diseases. Successful disease-control interventions in similar communities will need to engage with farmers' firsthand experiences and resource constraints, including the economic pressures that make selling sick animals or bypassing health documentation rational responses rather than deliberate negligence. Policy designs that acknowledge these constraints, and that support accessible veterinary services and realistic alternatives to natural bull breeding, are more likely to gain traction than those relying on enforcement alone. Understanding the local context is essential for obtaining accurate information about how disease risks evolve and how people in that environment mitigate these risks. Future research should investigate how interactions with formal animal health-care systems, including veterinary officers, vary by age and gender; how disease-control practices change across ecological zones in Kenya; and how longitudinal or ethnographic approaches can deepen understanding of the socio-cultural dimensions of livestock movement and farmer adaptation over time.

## Ethics statement

We received ethical clearance from the National Council for Science, Technology, and Innovation Committee (NACOSTI/P/23/25,811) and the Institutional Ethics and Review Board of ILRI-International Livestock Research Institute in Kenya (ILRI-IREC2023–52).

## Funding

This study did not receive any form of funding from third parties or any funding agencies to carry out this research study.

## CRediT authorship contribution statement

**Jackline Akoth Owili:** Writing – review & editing, Writing – original draft, Visualization, Project administration, Methodology, Investigation, Formal analysis, Data curation, Conceptualization. **Harro Maat:** Writing – review & editing, Writing – original draft, Supervision, Resources, Project administration, Methodology, Conceptualization. **Barbara van Mierlo:** Writing – review & editing, Writing – original draft, Supervision, Resources, Project administration, Conceptualization.

## Declaration of competing interest

The authors declare that they have no conflict of interest with the manuscript and its submission to the Journal of Veterinary and Animal Sciences.

## Data Availability

Data will be made available upon reasonable request to the first and corresponding author.
